# EEG Multiscale Complexity in Schizophrenia During Picture Naming

**DOI:** 10.3389/fphys.2018.01213

**Published:** 2018-09-07

**Authors:** Antonio J. Ibáñez-Molina, Vanessa Lozano, María. F. Soriano, José. I. Aznarte, Carlos J. Gómez-Ariza, M. T. Bajo

**Affiliations:** ^1^Department of Psychology, University of Jaén, Jaén, Spain; ^2^Department of Experimental Psychology, University of Granada, Granada, Spain; ^3^Hospital San Agustín, Jaén, Spain

**Keywords:** schizophrenia, EEG, non-linear analysis, multiscale lempel-ziv complexity, naming task

## Abstract

**Introduction:** Patients with schizophrenia show cognitive deficits that are evident both behaviourally and with EEG recordings. Recent studies have suggested that non-linear analyses of EEG might more adequately reflect the complex, irregular, non-stationary behavior of neural processes than more traditional ERP measures. Non-linear analyses have been mainly applied to EEGs from patients at rest, whereas differences in complexity might be more evident during task performance.

**Objective:** We aimed to investigate changes in non-linear brain dynamics of patients with schizophrenia during cognitive processing.

**Method:** 18 patients and 17 matched healthy controls were asked to name pictures. EEG data were collected at rest and while they were performing a naming task. EEGs were analyzed with the classical Lempel-Ziv Complexity (LZC) and with the Multiscale LZC. Electrodes were grouped in seven regions of interest (ROI).

**Results:** As expected, controls had fewer naming errors than patients. Regarding EEG complexity, the interaction between Group, Task and ROI indicated that patients showed higher complexity values in right frontal regions only at rest, where no differences in complexity between patients and controls were found during the naming task. EEG complexity increased from rest to task in controls in left temporal-parietal regions, while no changes from rest to task were observed in patients. Finally, differences in complexity between patients and controls depended on the frequency bands: higher values of complexity in patients at rest were only observed in fast bands, indicating greater heterogeneity in patients in local dynamics of neuronal assemblies.

**Conclusion:** Consistent with previous studies, schizophrenic patients showed higher complexity than controls in frontal regions at rest. Interestingly, we found different modulations of brain complexity during a simple cognitive task between patients and controls. These data can be interpreted as indicating schizophrenia-related failures to adapt brain functioning to the task, which is reflected in poorer behavioral performance.

**Highlights:**

- We measured classical and multiscale Lempel-Ziv Complexity (LZC_N_ and MLZC) of the EEG signal of patients with schizophrenia and controls at rest and while performing a cognitive task.

- We found that patients and controls showed a different pattern of brain complexity depending on their cognitive state (at rest or under cognitive challenge).

- Our results illustrate the value of the MLZC in the characterization of the pattern of brain complexity in schizophrenia on function of frequency bands.

- Nonlinear methodologies of EEG analysis can help to characterize brain dysfunction in schizophrenia.

## Introduction

Patients with schizophrenia suffer from cognitive impairments in a wide number of domains that include attention, language, working memory, episodic and semantic memory (Heinrichs and Zakzanis, 1998; Addington et al., 2003). However, many studies have shown evidence that the cognitive deficit that underlies schizophrenia is not generalized, but specific to some functions (e.g., semantic memory, working memory) (Cohen and Servan-Schreiber, 1992; Goldman-Rakic, 1994; Kerns and Berenbaum, 2002; Jonides and Nee, 2005). In addition, there have been many attempts to specify the brain dysfunctions related to the specific cognitive deficits by analyzing EEG signals. Electrophysiological studies have traditionally used methodologies of linear analyses, such as Event-Related Potentials (ERP) or power analysis (Kiang et al., 2007; Hughes et al., 2012). The absence of abnormalities of the raw EEG in patients with schizophrenia has led researchers to study quantitative parameters of EEG (QEEG). Spectral and coherence analyses of EEG are commonly used in the studies of QEEG abnormalities in schizophrenia (see Hughes and John, 1999).

Recently, new approaches to the study of EEG signals have been developed from non-linear system theories that can be helpful to understand brain dysfunctions associated with schizophrenia. Non-linear measures might render more adequate to reflect the complex, irregular and non-stationary behavior of neural processes. Some have suggested that non-linear approaches may be more powerful than classical lineal analyses to relate brain patterns of activation to cognition (e.g., Pereda et al., 2005; Klonowsky, 2009). EEG signals are the result of the non-linear combination of electrical activity generated by interacting oscillators from the cerebral cortex and other biological sources such as muscles. Consequently, the EEG signals have complex non-linear structures when looking at them in the time dimension. Most non-linear analyses have tried to quantify the complexity of EEG signals and to relate it to functional aspects of the neural networks. Thus, EEG complexity has been related to the integrity of neural connectivity, and with the number of distinct generators contributing to a given EEG signal (e.g., Lutzenberger et al., 1995). Hence, the more complex the signal is, the wider the distribution of cortical activation related to it (e.g., Mölle et al., 1999). Complexity is also related to the synchrony of oscillations of the generators. Synchronization between oscillators has been proposed as a general mechanism for information exchange within neural circuits (e.g., Engel et al., 2001; Fries, 2005). In general, it has been shown that synchrony is negatively related to complexity (Escudero et al., 2015; Ghanbari et al., 2015). While this relationship is far from being perfect (Ibáñez-Molina et al., 2018), highly synchronized signals (e.g., epileptic seizures) give rise to low complexity values (Radhakrishnan and Gangadhar, 1998). In sum, and although the exact meaning of complexity is still a matter of debate, complexity seems to be related to a number of variables: connectivity of neural networks, number of oscillators involved in the generation of signals, and synchrony of oscillations. Hence, for a given cognitive function, complexity reflects key functional aspects of the underlying neural sources. In general, high levels of complexity in the EEG recording indicate that the neural generators of the signal tend to be widely distributed and desynchronized. On the contrary, a low level of complexity indicates that the neural generators tend to be local and/or synchronized.

A number of complexity measures have been developed, some of which [the correlation dimension (D2), the Lyapunov exponent (L1), the Lempel-Ziv complexity (LZC), and the multiscale entropy analysis (MSE)] have been applied to EEGs from psychiatric patients (Sohn et al., 2010; Fernández et al., 2011; Bachiller et al., 2014). However, there are important differences among these methods. Thus, for example, while D2 and L1 are chaos-based estimates of complexity, LZC is based on algorithmic complexity, and MSE quantifies entropy over multiple time scales. More relevant, D2 and L1 require a large amount of EEG data, whereas LZC and the MSE are suitable for short and non-stationary time series.

Most of these measures quantify the degree of randomness or degrees of freedom of a system. Indeed, at a conceptual level, complexity has been often interpreted as irregularity, unpredictability, desynchrony or randomness (see Stam, 2005, for a review). However, it has been pointed out that complexity should not be equated to randomness, but to an intermediate state between randomness and order (Tononi and Edelman, 1998; Stam, 2005; Yang and Tsai, 2013). Yang and Tsai (2013) have proposed that brain complexity underlies the behavioral ability to adapt to the constantly changing environment. From this view, an abnormal brain complexity would give rise to either highly ordered or highly random behavioral patterns. Both regular and random patterns can be indicative of pathology and represent a deviation from complexity (Goldberger et al., 2002; Yang et al., 2015). This idea is supported by evidence showing lower values of brain complexity in some disorders (e.g., Alzheimer’s disease, Stam et al., 2009), whereas higher values of complexity are found in other disorders (i.e., schizophrenia) or during normal aging (Yang and Tsai, 2013).

Over the last years non-linear analyses have proven their utility to detect changes in brain complexity in some mental disorders (Yang and Tsai, 2013), with most studies focused on schizophrenia. (Fernández et al., 2011; Fernández et al., 2013). Some of these studies have found that patients with schizophrenia exhibit higher complexity than healthy controls in their EEG signals (Li et al., 2008; Takahashi et al., 2010; Fernández et al., 2011), although decreased complexity values have also been reported (Hoffmann et al., 1996; Lee et al., 2001; Akar et al., 2016). These apparently inconsistent findings might be explained by a number of confounding variables, such as the nature of the complexity estimates employed or the condition under which patients are tested (e.g., rest, closed eyes or active processing). Additionally, a relevant issue is that complexity is modulated by age. While complexity increases with age in healthy people, the opposite trend has been observed in patients with a mental disorder (Fernández et al., 2011; Méndez et al., 2012).

In a recent review, Fernández et al. (2013) proposed that three main variables seem to modulate EEG complexity in schizophrenia: medication, age, and symptomatology. Thus, increased complexity in schizophrenia is found in those studies that include young patients without medication and with a predominance of positive symptoms. In a previous study (Fernández et al., 2011), these authors reported increased complexity in patients with schizophrenia compared with a control group. However, whereas complexity positively correlated with age in the control group, patients with schizophrenia exhibited the opposite pattern (decreasing complexity with age). With regard to medication, some studies have shown that antipsychotics reduce complexity (as measured with MSE) in patients with schizophrenia (Takahashi et al., 2010), and that antidepressants reduce the usually high values of complexity in depression (Méndez et al., 2012).

Finally, EEG complexity also depends on the recording conditions. Several studies have reported that complexity increases in healthy participants while performing cognitive tasks (arithmetic, visual and reading tasks, see Stam, 2005, for a comprehensive review). On the contrary, Ibáñez-Molina and Iglesias-Parro (2014) have shown that EEG complexity is lower when healthy participants attend to visual or auditory stimuli than when they attend to their own thoughts (mind wandering). Furthermore, whether complexity increases or diminishes with cognitive demands might rely on the specific brain networks involved in the cognitive task.

In schizophrenia, most studies have focused on EEGs from patients at rest (Fernández et al., 2011), though exceptions exist (Kirsch et al., 2000; Li et al., 2008; Bachiller et al., 2014). Kirsch et al. (2000) compared EEG complexity (D2) of patients and controls at rest and while performing the continuous performance test (CPT). While they did not find differences between the two groups in the resting state condition, control participants showed a decrease in complexity when performing the cognitive task that was not observed in patients. According to the authors of the study, the healthy controls, but not the patients, were able to adjust their brain functioning to the task demands. However, because they recorded EEG from a unique electrode (in the Cz site), they were not able to explore changes in complexity on different brain regions under cognitive processing. Li et al. (2008) compared LZC of the EEG from patients with schizophrenia and depression with that of controls at rest and while performing a mental arithmetic task. Patients with schizophrenia showed higher LZC than controls at most electrodes. Both groups exhibited a decreased LZC during the task, although this decrease was smaller in the control group. More recently, Bachiller et al. (2014) compared spectral entropy (SE) between a resting condition and a task condition (auditory odd-ball task) in patients with schizophrenia and controls. SE quantifies the degree of disorder in a signal. They found that controls showed a decrease in entropy when performing the cognitive task, compared to rest, at parietal and central brain regions, whereas patients showed a reliable lower decrease than controls. Similar to Kirsch et al. (2000), Bachiller et al. (2014) failed to find differences in brain complexity between patients and controls in the resting state condition. On the contrary, Carlino et al. (2012) found a significant increase in EEG complexity (D2) during “active” conditions (eyes open, counting forward and counting backward conditions) compared to an eyes-closed resting condition, but only in the control group. At rest, however, the authors found greater complexity in patients than in controls. In sum, the evidence regarding EEG complexity while participants are performing cognitive tasks is mixed. It is possible that differences in the cognitive demands of the tasks, or in the non-linear measures used in different studies, underlie these divergences.

In the present study we aimed to gain further insight into how brain complexity changes under cognitive demands in patients with schizophrenia and healthy controls. To this end, we recorded EEGs from participants with schizophrenia and healthy controls at rest and while they performed a picture naming task. This cognitive task was selected for two reasons. First, it is a short and easy task wherein participants have to attend and name aloud visually presented stimuli. In addition, and more relevant, the picture-naming task has shown to be useful as a measure of semantic memory impairments in patients with schizophrenia (Soriano et al., 2008), which have been widely reported in this population (Manschreck et al., 1988; Kerns and Berenbaum, 2002). Specifically, here we compared EEG complexity in patients and controls in a resting state condition (seating and with open eyes) and while they were performing the naming task. Complexity was estimated with the classical Lempel-Ziv complexity analysis (LZC_N_) and the modified LZC to measure different frequency bands (Ibáñez-Molina et al., 2015). This modified measure was termed Multiscale Lempel-Ziv complexity (MLZC) and we selected it because of its several advantages over other non-linear measures: namely, it can be applied to short time series and non-stationary and noisy signals. In addition, the MLZC measure allows for the exploration of the signal at different time scales. Previous results have shown that the classical LZC neglects rapid components of the EEG signals (Ibáñez-Molina et al., 2015; Kalev et al., 2015). The Multiscale LZC, however, allows for a better characterization of EEG complexity in different frequency bands. Most rapid components of the EEG signals reflect local functional configurations in the cortex, whereas slow oscillations reflect more long-range cortical interactions (Buzsáki and Draguhn, 2004). However, a word of caution is necessary regarding the causal role of fast and slow rhythms, since slow oscillatory activity could reflect the long-range coordination of faster components or the operation of a single mechanism that generates the specific rhythm. Despite this, the MLZC permits a better characterization of the signal in terms of its oscillatory components and, because it is more sensitive to rapid rhythms, it might serve as a more suitable tool to detect local neural interactions than the classical LZC.

Based on previous results (Li et al., 2008; Takahashi et al., 2010; Fernández et al., 2011), we expected to find higher EEG complexity in patients, compared to controls, in the rest condition. We also hypothesized that complexity would vary in healthy controls while performing the naming task. More interesting, and given that patients with schizophrenia usually show poorer performance in naming tasks (Soriano et al., 2008), we aimed to explore whether their EEG complexity was modulated by the fact of performing the cognitive task. Finally, we aimed to examine through the MLZC whether differences in complexity between patients and controls depend on specific components (slow or rapid) of the EEG signal.

## Materials and Methods

### Participants

The patients group was composed of 18 participants attending the Mental Health Day Hospital of the St Agustín Hospital in Linares. In their clinical record, they were diagnosed with schizophrenia, schizophreniform or schizoaffective disorder according to DSM-IV criteria. The patients’ diagnosis was confirmed through a clinical interview performed by the psychiatrist or clinical psychologist in charge of the patient. In addition, the Spanish version (Peralta and Cuesta, 1994) of The Positive and Negative Syndrome Scale (PANSS) (Kay et al., 1987) was used to evaluate patients’ current clinical state; and some additional demographic information was obtained (see **Table 1**). At the time of testing, all patients were taking antipsychotic medications with good compliance. All the patients were receiving atypical antipsychotics, usually risperidone, olanzapine, or clozapine. Before participating, they were informed of the task and study and asked to sign informed consent forms in accordance with Ethical Committee of the Hospital.

**Table 1:** Demographic and clinical characteristics of the study sample means (and standard deviations).

	Patients (*n* = 18)	Control (*n* = 17)
Age, years	35.26 (8.96)	29.6 (9.42)
Females	3	6
Education	2.39 (0.21)	2.59 (0.22)
Illness duration, years	13.21 (9.47)	-
GAF	45.44 (17)	-
PANNS		
Positive	14.9 (7.9)	-
Negative	17.16 (6)	-
General	34.05 (8.4)	-


*^∗^Groups did not differ significantly on any of the demographic characteristics (*p* < 0.05).*

The control group was composed of 17 healthy adult participants. They were recruited from the family members of the clinical and research staff of the Unit. Care was taken that none of the control participants had a history of psychotic disorders, or family members with psychotic disorders. In addition, none of the participants, control or patients, had a history of substance use disorders, neurological illness, head trauma, or mental retardation. There were no significant differences between patients and controls in age or educational level (see **Table 1**).

The number of participants per group was decided on the basis of the sample sizes considered in previous studies using EEG recordings on schizophrenic patients (e.g., Sabeti et al., 2009; Carlino et al., 2012).

### Task

Eighty black and white simple pictures were employed as targets. Three additional pictures were used for practice. The pictures were selected from the norms of Puerta-Melguizo et al. (1998). The order of the pairs was randomized. The STIM^2^ software was employed to create the task. Each trial consisted of a sequence of stimuli, which appeared in the center of a computer screen. First, a mask was presented for 500 ms and then the picture target was presented for 100 ms, the mask for another 14 ms, and finally a tone that signaled the participant to respond. Thus, responses were delayed, in order to avoid the influence of vocal movements on the EEG recording. Participants were instructed to look at the center of the screen and to name the pictures as soon as they heard the tone. The experimenter registered the participants’ responses. The task took about 10 min.

### EEG Recording

EEG data were obtained with a 36 Ag/Ag Cl electrodes cap (QuikCap), and they were recorded with a sampling frequency of 1000 Hz (22 bits). EEG was recorded at rest and while the naming task was being performed. Participants were always seated in a chair opposite the computer screen. A Neuroscan SynAmps 32-channel amplifier was used for data acquisition. EEG data were applied a band-pass filter with cut-off frequencies of 1 and 30 Hz. The reference electrode was the left mastoid. The influence of eye movements on the EEG signal was eliminated through ERPlab. Facial movements were recorded through 4 electrodes and segments that included them were eliminated. EEG segments corresponding to errors in the naming task were also excluded from the analysis. Electrodes impedance was maintained below 5 kΩ for all participants. The rest segments were selected from each participant right before the task with a length of 5 × 10^4^ ms. EEG segments from the task of 2 × 10^3^ ms were extracted after the appearance of each trial. They were selected so that the influence of the verbal responses was not included.

### Analyses

#### Behavioral Data

A response was considered an error when the participants stuttered or hesitated in naming the target, or they misnamed or failed to name the target. We compared error percentages in patients and controls. Naming times were not analyzed, since vocal responses were delayed in order to avoid muscle influences on the EEG recording.

#### Classical LZC and Multiscale LZC

Lempel-Ziv Complexity measures complexity as defined by Kolmogorov (1965); namely, the number of bits of the shortest computer program that can generate the analyzed time series. Thus, LZC tests the randomness of a sequence by searching for patterns in the series (Lempel and Ziv, 1976). Recent studies have presented it as an effective tool in analyzing biomedical signals (Zozor et al., 2005), and in fact it has been widely used to characterize the EEG of several mental and neurological disorders (Nagarajan, 2002; Aboy et al., 2006). For the present study we employed the classical LZC (LZC_N_) and the MLZC measure introduced by Ibáñez-Molina et al. (2015). The original LZC measure estimates the complexity of a time series by a binarization process in which the signal is transformed into a binary sequence by using its median as a threshold. It has been shown that this based-on-median binarization neglects fast components of the EEG signals (Ibáñez-Molina et al., 2015). The MLZC can be seen as a generalization of the original LZC because it uses multiple thresholds for binarization. Thresholds are median-based smoothed versions of the original signals. By increasing the width of the window used for the smoothing, the new versions include less and less fast components. Thus, when used as thresholds for binarization they capture the missing fluctuations in the original series. Hence, this procedure allows us to capture signal variations at different time scales that make it possible to obtain a spectrum of complexity ranging from fast to low rhythms. That is, the MLZC considers both temporal and spectral information from the EEG signals and, consequently, it permits the evaluation of the complexity of the different brain rhythms and the detection of complexity variations in a specific oscillatory band. It is also possible to relate a specific threshold of binarization to a particular frequency band on the basis of the sampling rate of the signal, so that the width of the smoothing procedure can be associated with a particular frequency. Thus, for example, to capture a rhythm of 1 Hz with a sampling rate of 1000 Hz, we need at least a smoothing with a window length of 1000 points.

Formally, a 0-1 sequence {*p*(*n*)} = *s*(1),*s*(2)…*s*(*N*), was created by comparison of each data point *x*(n) in the series with its *T_d_* in the following way:

(1) $$s(n)=\left\{ \begin{array}{c} 0\,\;if\,\;x(n)<T_{d}\\ 1\,\;if\,\;x(n)\geq T_{d} \end{array}\right.$$

The first binary sequence was constructed using the median of the entire signal as *T_d_* (*T_dN_*). The other binarizations were created using smoothed versions of the signal as *T_d_*s. Each data point *x*(n) had an unique *T_dw_*(n) which was calculated by:

(2) $$T_{dw}(n)=median(x(n-\frac{w_{k}-1}{2}),....,x(n),...,x\left(n+\frac{w_{k}-1}{2}\right)),n=1+\frac{w_{k}-1}{2},.....,N-\frac{w_{k}-1}{2}$$

where W = [w_k_,…,w_m_], *k* = 1,…,m is the vector that contains window lengths of the smoothing procedure.

In order to obtain the LZC spectrum of all {*x*(*n*)}, each *P*_w_(n) was explored according to the following steps:

(a) EEG segments were analyzed using segments of 2×10^3^ms and averaged for each experimental condition. In the rest condition, long segments (5 × 10^4^ ms) were analyzed using a moving window procedure. The moving window length was 2 × 10^3^ ms with an overlap of 2 × 10^2^ ms. In the task condition, EEG segments were time locked to stimulus onset for each trial. Hence, at task, a total of 80 segments of 2 × 10^3^ms were analyzed for each participant and then averaged to obtain a final value of MLZC.
(b) LZC was calculated for each window by means of a complexity counter *C*_w_(n). During a left to right scan of a given binary sequence, *C*_w_(n) increased by one unit every time a new subsequence of consecutive characters was encountered.
(c) Each LZC_w_ was obtained when *C*_w_(n) values were normalized with

(3) $$LZC=\frac{C_{w}\left(n\right)}{\frac{n}{\log_{2}n}}$$

where the sub index w indicates the window length of the smoothing that produced *P*_w_(n). Note that LZC_N_ will refer to the median based LZC.
(d) The final LZC_w_ value of each signal was calculated by the average of all values obtained with the moving window procedure.

## Results

Analyses of the behavioral accuracy data indicated that patients committed more errors (10%, *SD* = 7.1) than controls (6%, *SD* = 3.4), though the effect only approached to statistical significance [*F*(1,33) = 7.47; *MSE* = 73.73; *p* = 0.07].

**FIGURE 1:**
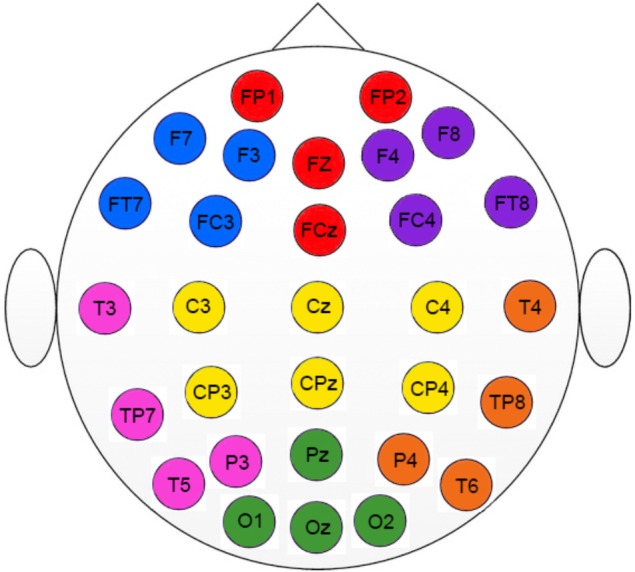
Regions of interest used in the study (ROI). Colors indicate the electrodes for each specific ROI. Left frontal (blue); Frontal (red); Right frontal (purple); Left temporal-parietal (pink); Central (yellow); Right temporal-parietal (orange); Parietal-occipital (green).

**FIGURE 2:**
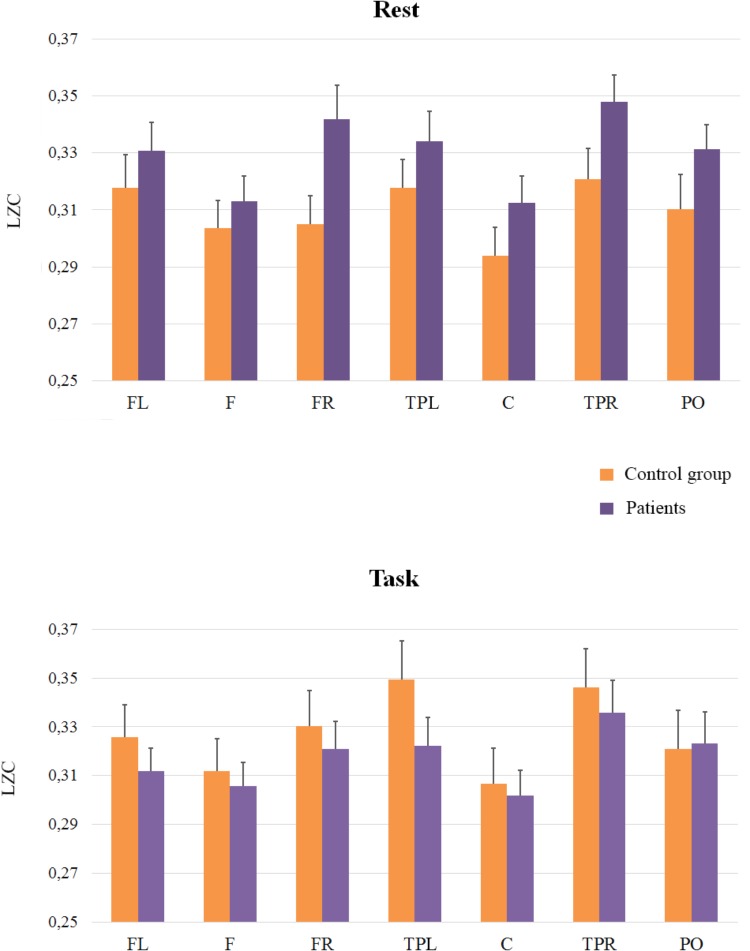
Means and SE (error bars) of Classical LZC for each ROI. Labels in the x-axis indicate each ROI: Left frontal (LF); Frontal (F); Right frontal (RF); Left temporal-parietal (TPL); Central (C); Right temporal-parietal (TPR); Parietal-occipital (PO).

Regarding electrophysiological data, electrodes were grouped in seven Regions Of Interest (ROI, see **Figure 1**). We conducted three mixed ANOVAs with Group as the between participants factor, and Cognitive State (rest vs. task) and ROI (1: left frontal 2: frontal 3: right frontal 4: left temporal-parietal 5: central 6: right temporal-parietal 7: parietal-occipital, see **Figure 1**), as within-participant variables. Each ANOVA was conducted on a specific range of scales. The first analysis was performed on the classical LZC_N_ measure (see **Figure 2**). The results of this analysis showed a reliable main effect of ROI, [*F*(6,198) = 13.43; *MSE* = 0.001; *p* < 0.01]; complexity was significantly lower in medial Central and Frontal regions than it was in the rest of regions. The effect of group did not reach statistical significance (*F* < 1). However, more importantly, we found a significant Group × Cognitive State × ROI interaction [*F*(6,198) = 2.18; *MSE* = 0.0003; *p* < 0.05]. In order to examine this second-order interaction, we analyzed separately the effects of ROI and Cognitive State in each group. We found a reliable ROI × Cognitive State interaction in the control group [*F*(6,96) = 26.9; *MSE* = 0.0008; *p* < 0.05], which showed that control participants exhibited greater complexity in Left-Temporal-Parietal regions while performing the task than at rest, [*F*(1,33) = 6.12; *MSE* = 0.001, *p* < .05]. On the contrary, the ROI x Cognitive State interaction did not reach statistical significance in patients (*F* < 1): there were no significant differences in complexity between rest and task in the patients group.

In addition, we compared the groups at rest and during task performance: planed comparisons showed that in the rest condition complexity was higher in patients than in controls in Right-Frontal [(*F*(1,33) = 5.53; *MSE* = 0.002; *p* < 0.05)] and in Right-Temporal-Parietal regions, even though this latter effect was statistically marginal, [*F*(1,33) = 3.53; *MSE* = 0.002; *p* = 0.06]. During task performance, however, there were no significant differences between patients and controls (*F* < 1).

**FIGURE 3:**
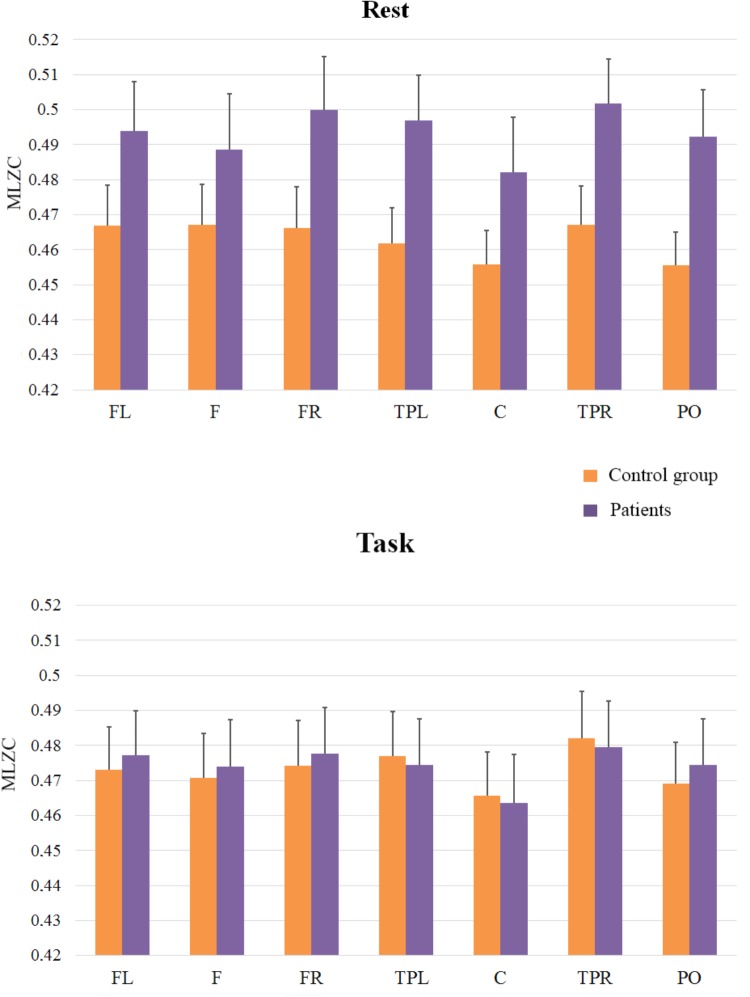
Means and SE (error bars) of fast rhythms MLZC for each ROI. Labels in the x-axis indicate each ROI: Left frontal (LF); Frontal (F); Right frontal (RF); Left temporal-parietal (TPL); Central (C); Right temporal-parietal (TPR); Parietal-occipital (PO).

The second ANOVA was carried out to investigate the complexity of the signals in scales ranging from LZC_21_ to LZC_101_ (frequency bands > 10 Hz), since we aimed to evaluate the complexity predominantly associated with fast rhythms and low amplitudes (See **Figure 3**)_._ As in the previous analysis, the main effect of ROI was reliable [*F*(6,198) = 7.25; *MSE* = 0.0003; *p* < 0.051], and it reflected that complexity was lower in Central Region than in the rest of regions. The effect of Group [*F*(1,33) = 1.22; *MSE* = 0.0257; *p* = 0.28] and the interactions between ROI and Group (*F* < 1), and Cognitive State and Group [*F*(1,33) = 2.1; *MSE* = 0.0127; *p* = 0.16] did not reach significance, but the interaction of Group × Cognitive State x ROI, *F*(6,198) = 2.1; *MSE* = 0.0001; *p* = 0.05 did. In order to examine this second-order interaction, we analyzed the effects of ROI and Cognitive State in each group. Again, we found a significant ROI × Cognitive State interaction in the control group [*F*(6,96) = 2.8, *MSE* = 0.0002; *p* < 0.05], while the ROI × Cognitive State interaction was not significant in the patients group (*F* < 1). In addition, planned comparisons showed higher levels of complexity in the rest condition for patients than for controls in Left-temporal-parietal [*F*(1,33) = 4.41; *MSE* = 0.002; *p* < 0.05], and Right-temporal-parietal [*F*(1,33) = 4.13; *MSE* = 0.002; *p* = 0.05] sites, while no effects were found in the task condition (all *p*s > 0.5).

**FIGURE 4:**
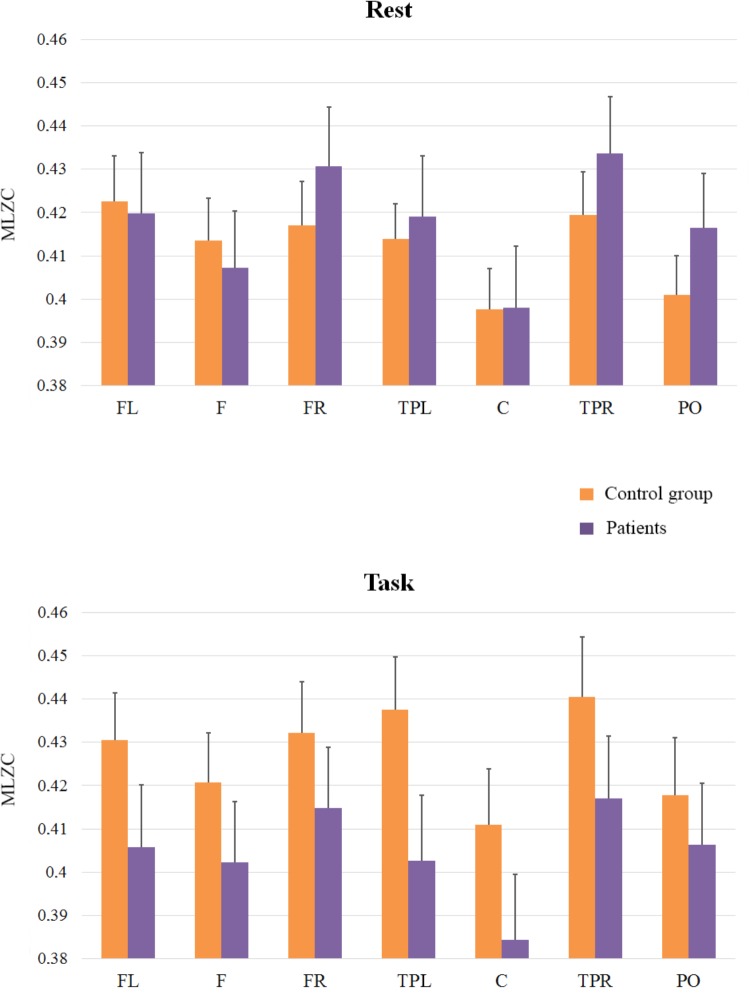
Means and SE (error bars) of slow rhythms MLZC for each ROI. Labels in the x-axis indicate each ROI: Left frontal (LF); Frontal (F); Right frontal (RF); Left temporal-parietal (TPL); Central (C); Right temporal-parietal (TPR); Parietal-occipital (PO).

The third analysis (see **Figure 4**) was applied to scales from LZC_121_ to LZC_201_ to explore slow rhythms (frequency bands in an approximate range of 5–8 Hz). It revealed a main effect of ROI [*F*(6,198) = 15.95; *MSE* = 0.0004; *p* < 0.01] and a similar pattern of complexity across regions to that found in previous analyses; The effect of Group (*F* < 1), and the interactions between ROI and Group [*F*(6,198) = 1.80; *MSE* = 0.0004; *p* = 0.10], and Cognitive State and Group [*F*(1,33) = 2.07; *MSE* = 0.0117; *p* = 0.16] did not reach significance, but the interaction of Group x Cognitive State x ROI was significant [*F*(6,198) = 2.19; *MSE* = 0.0002; *p* < 0.05]. Interestingly, the pattern of results differed from that of the fast scales, indicating that the complexity of EEG signals was similar in patients and in controls in the rest condition (*F* < 1), but complexity in patients tended to be lower than in control participants in the task condition. Although this tendency did not reach statistical significance, it was marginally significant at the central region [*F*(1,33) = 3.18; *MSE* = 0.003; *p* = 0.08].

Finally, and because the ANOVAs failed to capture the rest to task changes in complexity in patients and controls (see **Figures 2**, **4** in nearly all the regions), we explored this general pattern by categorizing the rest vs. task changes as ‘up’ (Task-Rest > 0) or ‘down’ (Task-Rest ≤ 0), and then performing a non-parametric chi-squared test on each region. The results of this analysis (see **Table 2**) revealed that the differences between the groups in complexity changes from rest to task are especially evident in fast rhythms.

**Table 2:** Non-parametric analyses of complexity changes from Rest to Task in patients vs. controls on each region of interest.

	LZC_N_ χ^2^ (gl = 1), *p*-value	LZC_21-101_ χ^2^ (gl = 1), *p*-value	LZC_121-201_ χ^2^ (gl = 1), *p*-value
FL	3.54, 0.06	1.37, 0.24	2.44, 0.12
F	4.80, 0.03^∗^	1.37, 0.24	0.77, 0.38
FR	4.80, 0.03^∗^	3.44, 0.06	1.45, 0.23
TPL	0.31, 0.58	3.73, 0.05	1.70, 0.19
C	0.72, 0.39	6.41, 0.01^∗^	3.73, 0.05
TPR	2.62, 0.10	6.56, 0.01^∗^	0.72, 0.39
PO	0.70, 0.40	4.06, 0.04^∗^	2.91, 0.09


*^∗^Groups differed significantly in the Task – Rest qualitative variable (*p* < 0.05). Task vs. Rest complexities were characterized by a categorical variable with the value ‘up’ in the case that the transition from rest to task was positive, and the value ‘down’ if the transition was negative. Differences between the control and patients groups were analyzed for each region of interest by χ^2^ tests.*

## Discussion

EEG complexity is being increasingly used to explore brain dynamics in healthy and pathological states, since complexity indexes might more adequately reflect the complex, irregular, non-stationary behavior of neural processes than more traditional ERP measures. The present work aimed to explore possible differences in EEG complexity between patients with schizophrenia and controls under conditions involving different cognitive demands. Overall, our results showed two important patterns: (1) patients exhibited higher complexity in frontal regions than control participants at rest; and (2) while control participants showed an increment in complexity from rest to task, there were no reliable differences in complexity between rest and task in the patients group.

Regarding the higher complexity in frontal regions for patients at rest, our findings are in accordance with those from most recent studies with patients with features similar to the ones displayed by our patients’ sample. Thus, although there are some divergent results (Li et al., 2008; Takahashi et al., 2010; Fernández et al., 2011; Akar et al., 2016), higher complexity has been mainly observed in young, drug-naive patients with active symptomatology, whereas lower complexity than controls has been observed in studies with medicated chronic patients (Fernández et al., 2013). Because our patients were recruited from a Mental Health Day Hospital, although they were medicated, most of them were young adults with active psychotic symptoms (see **Table 1**). In addition, higher complexity at rest has also been found in other mental disorders such as depression (Méndez et al., 2012), while lower complexity has been found in Alzheimer Disease (AD) (Jeong, 2004; Stam et al., 2009). As we mentioned, higher complexity values would reflect more and more widely distributed neural nodes oscillating at a lower synchrony. Hence, high complexity in schizophrenia (and other severe mental disorders as depression) could be indicative of isolation or disconnection of brain nodes (Friston et al., 1995) as well as disorganization of spiking activity (Takahashi et al., 2010).

The finding of lower complexity in patients with schizophrenia in some of previous studies could be due to the elevated requirements of some measures (classical measures as D2 and L1 require stationary dynamical systems). Distinct tolerance to noise or requirements related to length of time-series are other variables that could explain the lack of agreement in results. In the context of mental disease, Sabeti et al. (2009) compared the discriminative power of several measures and found that Higuchi fractal dimension, Lempel-Ziv complexity and Entropy indexes were the most informative in discriminating between patients with schizophrenia and controls.

The second and more remarkable finding in the present study is that we observed (with the classical LZC_N_ measure) an increase in EEG complexity in Left-Temporal-Parietal regions during task performance only in controls, with the group of patients showing comparable complexity at rest and during task performance. Although it had been suggested that changes in complexity during cognitive processing might depend on the specific brain regions involved in the task (Elbert et al., 1994), research regarding this modulation has been scarce. In the present study the cognitive task was a visual naming task. Although it might seem low-demanding at first sight, picture naming involves a number of cognitive processes including visual perception, semantic memory and phonological retrieval (Race and Hillis, 2015). Studies employing functional neuroimaging or lesion data converge in the idea that semantic memory is generally dependent on the left hemisphere, specifically on ventral and lateral regions of the posterior temporal lobe (Chao et al., 1999; Hamberger, 2015; Race and Hillis, 2015). The lack of observable changes in brain complexity during the task in patients was also associated with impairment in performance in the naming task in patients. Together, these findings would support the idea that patients encountered difficulties to adapt their brain functioning to the task demands.

In addition, the use of the MLZC provides relevant data regarding differences in complexity between patients and controls in fast and slow components of the EEG. As we mentioned, the classical LZC neglects rapid components of the EEG signals (Ibáñez-Molina et al., 2015; Kalev et al., 2015). In contrast, the Multiscale LZC, allows for a better characterization of EEG complexity in different frequency bands. A study by Yang et al. (2013) has also spotlighted the importance of frequency bands in the estimation of complexity in AD. In this study, they found that increased severity of AD was associated with decreased MSE complexity as measured by short-time scales, but with increased MSE complexity as measured by long-time scales. In a similar vein, Kalev et al. (2015) have shown that MLZC is able to capture differences in complexity between patients with depression and controls in the high frequency scales, whereas the classical LZC did not differentiate between the groups because it underestimated high frequency components of the EEG signal. Our results also illustrate the value of the MLZC in the estimation of complexity in different frequency bands. While the classical LZC estimate indicated that patients exhibited higher values of complexity at rest in some brain regions, separate analyses for rapid and slow scales pointed to a more complex pattern of results. Specifically, patients showed higher values of complexity during rest only for fast rhythms. On the contrary, control participants tended to present higher values of LZ during the task in slow rhythms. Although the functional meaning of these results is not evident to us, they support the idea that complexity should be separately assessed for different rhythms. To fully understand complexity for rapid and slow oscillatory rhythms, there are two aspects to consider: (1) the more complex a signal is, the more variability it exhibits, and (2) while the variability for fast rhythms reflects local functional configurations in the cortex, for slow oscillations complexity captures more long-range cortical interactions (Buzsáki and Draguhn, 2004). Hence, one could speculate that fast oscillations reflecting local dynamics of neuronal assemblies are more heterogeneous for patients at rest. This might result from an irregular by-default functioning at a local level. On the contrary, the higher complexity of the slow rhythms in controls might result from a more flexible establishment and switching between a large variety of long range cortical interactions directed to adapt themselves to the task at hand. Finally, the non-parametric analyses of rest-task changes showed that differences between patients and controls from rest to task were especially evident in fast frequencies, which is in line with the findings by Kalev et al. (2015) in depression. These results could be indicating that differences between patients and controls in cognitive functioning would rely more on local neural configurations than on the dynamics of whole-brain networks. The fact that the non-parametric analyses revealed differences between patients and controls in parietal and occipital electrode locations in fast scales, could be interpreted as reflecting abnormal processing in primary visual areas. This finding is in line with experiments showing that schizophrenic patients exhibit abnormal beta-gamma induced rhythm during visual perception (Uhlhaas et al., 2006; Uhlhaas and Singer, 2010; Grützner et al., 2013). However, we should note that this explanation is tentative and the present results are novel and need replication.

Some limitations of the study need to be considered. First, and most important, the small sample prevented us from analyzing the relationship between demographic and symptomatic characteristics of patients and EEG complexity. As previously mentioned, age and symptomatology influence complexity measures (Fernández et al., 2013). Second, all patients were on antipsychotic treatment, which could have impacted on their EEG patterns. Finally, we evaluated brain complexity of participants while performing a naming task. Cognitive tasks vary in a number of variables: processing system (attention, language, memory…), perceptual domain (visual, auditory…), and difficulty, among others. Therefore, it would be highly speculative to extrapolate results from a visual naming task to other cognitive tasks. Future research should address how changes in brain complexity are modulated by cognitive demands.

## Ethics Statement

This study was carried out in accordance with the ethical standards of the research committee of St Agustin Hospital and with the 1964 Helsinki declaration. The protocol was approved by the committee of St Agustin Hospital and all subjects gave written informed consent.

## Author Contributions

MS, MB, JA, CG-A, and AI-M made a substantial, direct and intellectual contribution to the main hypothesis and design of the experiment. VL and AI-M conducted the experiment and analyzed the EEG data sets. All authors participated in the preparation of the manuscript.

## Conflict of Interest Statement

The authors declare that the research was conducted in the absence of any commercial or financial relationships that could be construed as a potential conflict of interest.
